# Inhibition of cytochrome P450 3A by acetoxylated analogues of resveratrol in *in vitro* and *in silico* models

**DOI:** 10.1038/srep31557

**Published:** 2016-08-17

**Authors:** Loai Basheer, Keren Schultz, Zohar Kerem

**Affiliations:** 1 Institute of Biochemistry, Food Science and Nutrition, The Robert H. Smith Faculty of Agriculture, Food and Environment, The Hebrew University of Jerusalem, P.O. Box 12, Rehovot 76100, Israel

## Abstract

Many dietary compounds, including resveratrol, are potent inhibitors of CYP3A4. Here we examined the potential to predict inhibition capacity of dietary polyphenolics using an *in silico* and *in vitro* approaches and synthetic model compounds. Mono, di, and tri-acetoxy resveratrol were synthesized, a cell line of human intestine origin and microsomes from rat liver served to determine their *in vitro* inhibition of CYP3A4, and compared to that of resveratrol. Docking simulation served to predict the affinity of the synthetic model compounds to the enzyme. Modelling of the enzyme’s binding site revealed three types of interaction: hydrophobic, electrostatic and H-bonding. The simulation revealed that each of the examined acetylations of resveratrol led to the loss of important interactions of all types. Tri-acetoxy resveratrol was the weakest inhibitor *in vitro* despite being the more lipophilic and having the highest affinity for the binding site. The simulation demonstrated exclusion of all interactions between tri-acetoxy resveratrol and the heme due to distal binding, highlighting the complexity of the CYP3A4 binding site, which may allow simultaneous accommodation of two molecules. Finally, the use of computational modelling may serve as a quick predictive tool to identify potential harmful interactions between dietary compounds and prescribed drugs.

Cytochrome P450 enzymes (P450s) are very important for the metabolism of a wide range of endogenous compounds (e.g., steroid hormones, lipids, bile acids), as well as xenobiotics including drugs, environmental pollutants and dietary products^1^,^2^,^3^,^4^. Among the P450s, CYP3A4 is the main enzyme involved in drug metabolism. It is involved in the metabolism of over 50% of marketed drugs that rely on metabolic elimination^5^. Potential interactions between new molecules and CYP3A4 are routinely assessed during the early stage of drug development^6^,^7^. CYP3A4 accounts for up to 30% of the total cytochrome P450 (CYP) protein content of the human liver and is also expressed in the human small intestine, prostate, kidney, lungs, and brain^4^,^8^,^9^. The active site of a substrate-free CYP contains a heme prosthetic group, in which the iron is anchored by the four bonds of the heme group, the fifth proximal ligand of a conserved cysteine and a water molecule as the sixth distal ligand^3^. Like most other CYPs, it catalyzes a monooxygenase reaction (i.e., the insertion of one atom of oxygen into an organic substrate while another oxygen atom is reduced to water)^10^. The substrate chemical characteristics and the preferred position of insertion change from one CYP enzyme to another and are used for the classification of the CYP superfamily, as well as the structural homology^1^,^11^,^12^,^13^,^14^.

*t*-Resveratrol (trans-3,5,4′-trihydroxystilbene) is a polyphenol found in grape skin and red wine. The ability of resveratrol to inhibit CYP3A4 both *in vitro* and *in vivo* has been well established and it has been suggested that resveratrol may act as an irreversible, mechanism-based inactivator of this enzyme^15^,^16^,^17^,^18^,^19^,^20^. This mechanism-based inhibition occurs when a CYP3A4 substrate/inhibitor forms a reactive intermediate at the CYP3A4 active site, leading to enzyme inactivation by modification of the heme or the apoprotein^15^,^21^,^22^. Resveratrol exhibits high membrane permeability and is considered a class-II compound in the Biopharmaceutical Classification System (BCS)^23^. However, resveratrol has a low bioavailability (less than 1%) due to extensive first-pass metabolism by CYP3A4 in the intestine and the liver, which is extended by the enterohepatic recirculation of its glucuronide and sulfate metabolites^24^,^25^. Recently, it was reported that resveratrol sulfates are deconjugated by steroid sulfatase to free resveratrol *in vitro* and *in vivo*, allowing resveratrol sulfates to act as an intracellular reservoir for resveratrol^26^. Clinical and rat trials have found that administration of resveratrol increases the area under curve of several drugs^18^,^19^,^27^. Apparently, ingesting large amounts of resveratrol might increase the bioavailability of many drugs, as well as the risk of toxicity of drugs that undergo extensive first-pass metabolism by CYP3A4^20^.

A study of the effect of lipophilicity on the interactions of stilbenes with CYP3A4 revealed that methoxy-stilbenes have lower IC_50_ values and greater affinity for CYP3A4 than *t*-resveratrol or its glucosides^17^. The positive correlation between a molecule’s lipophilicity and its potential interaction with CYP3A4 has been further supported by QSAR studies^28^,^29^,^30^. In addition, the importance of potential hydrogen bonding in determining the nature of interactions with CYP3A4 has been proposed in previous works^14^,^31^,^32^,^33^,^34^. Substrate pharmacophore studies demonstrate a requirement for two hydrogen bond (H-bond) acceptors, one H-bond donor and one hydrophobic region^34^. *t*-Resveratrol contains three hydroxyl group (OH), which may act simultaneously as both H-bond donors and H-bond acceptors^35^,^36^. In a previous work, we showed that synthesizing resveratrol aldehyde to add a H-bond acceptor leads to the loss of several interactions at the binding site^37^. In the present work, using the predicted docking energy, we have designed novel acetoxy-stilbenes (Fig. 1), in an effort to identify structural determinants that increase the inhibition of CYP3A4, through an increase in either lipophilicity or the addition of H-bond acceptors. Previous works have suggested a high structural similarity between the human CYP3A4 and the isozymes CYP3A1 and CYP3A2 which are expressed predominantly in the rat liver^38^. It has also been calculated that CYP3A1 and CYP3A2 are 88% identical, and both share 72% and 73% protein homology to the human CYP3A4, respectively^14^. Using two *in vitro* models presenting different isozymes of CYP3A, i.e., human CYP3A4 in Caco2/TC7 cell culture, and CYP3A in rat liver microsomes (RLM), and an *in silico* docking model allowed us to define structural components required for a compound to potently inhibit CYP3A4.

## Results

### Testosterone metabolism in the Caco-2/TC7 cell line

**Figure 2:**
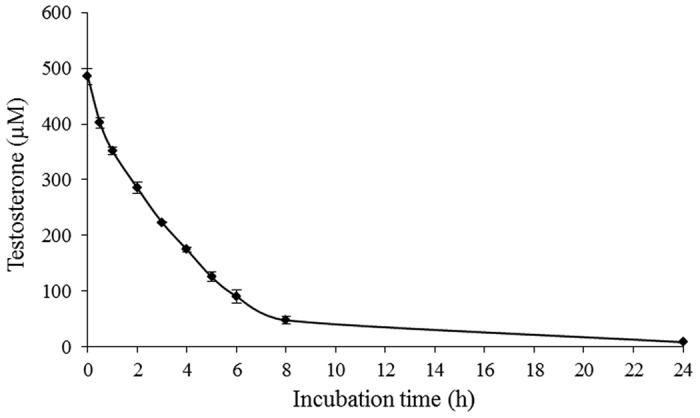
Metabolism of testosterone in the Caco-2/TC7 cell line. Data are means ± S.E. of three replicates.

The oxidation of testosterone by CYP3A4 was followed by the measurement of the residual testosterone in the reaction medium during incubation with Caco-2/TC7 cells (Fig. 2). Under the experimental conditions, a linear reaction rate was observed during the first 6 h of the incubation. Testosterone was quantified using HPLC analysis and a calibration curve.

### Inhibition of CYP3A4-mediated testosterone metabolism in the Caco-2/TC7 cell line by acetoxy stilbenes

**Figure 3:**
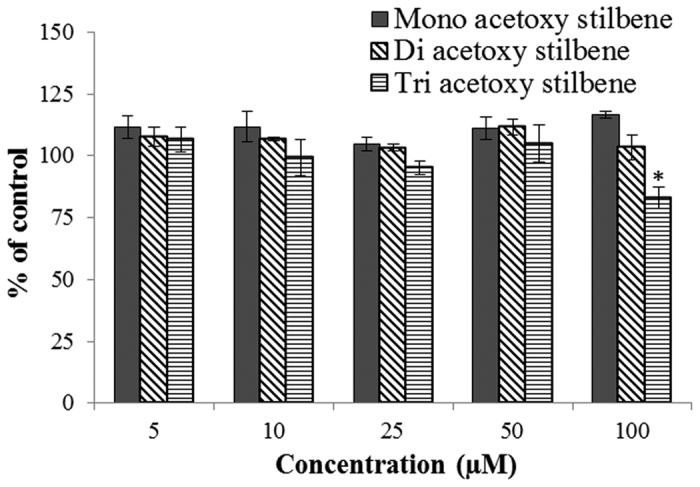
Caco-2/TC7 cell viability when different concentrations of mono/di/tri-acetoxy resveratrol were present in the medium. Data are means ± S.E. of five replicates. Asterisks indicate significant differences at *P *< 0.05, as determined by ANOVA followed by the Tukey-Kramer test.

The effects of MAR, DAR and TAR on the viability of Caco-2/TC7 cells were measured as described. None of the compounds caused a reduction of Caco-2/TC7 cell viability when applied up to a level of 100 μM, except for 100 μM of TAR, which caused a minor reduction in the cells proliferation (Fig. 3). The concentration of 100 μM was selected as the highest concentration to be included in the later experiments, considering the slight reduction in cell viability caused by 100 μM TAR.

**Figure 4:**
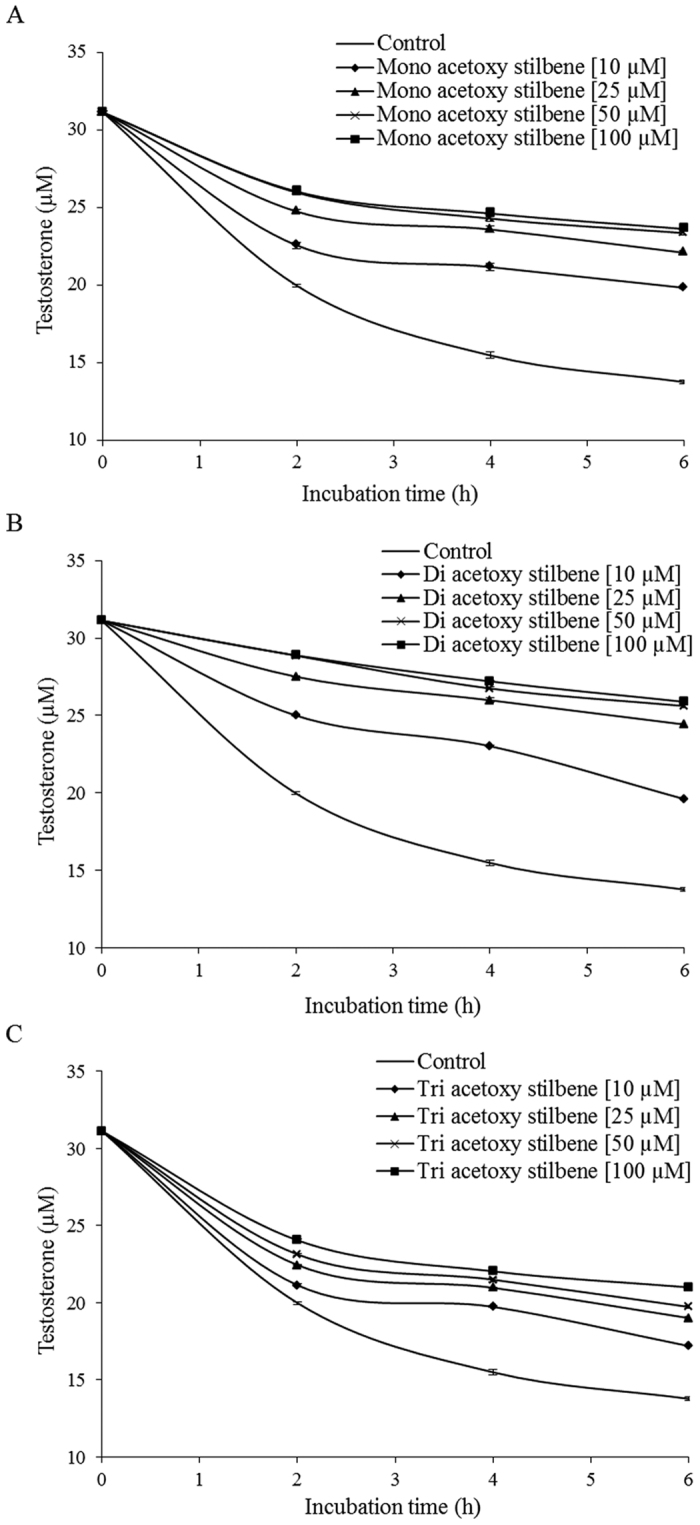
Inhibition of testosterone metabolism in Caco2/TC7 cells by different concentrations of mono-acetoxy stilbene (**A**), di-acetoxy stilbene (**B**) and tri-acetoxy stilbene over incubation times compared to control. Data are means ± S.E. of three replicates.

**Figure 5:**
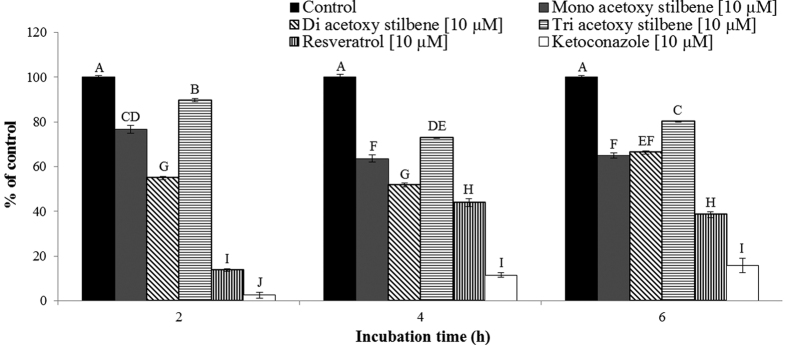
Inhibition of testosterone metabolism in Caco2/TC7 cells by the acetoxy analogues, resveratrol and ketoconazole, at a concentration of 10 μM, as compared to controls over time. Results are expressed as the percentage of the activity of the control for each incubation time. Data are means ± S.E. of three replicates. Different letters indicate significant differences at *P *< 0.05, as determined by ANOVA, followed by the Tukey-Kramer test.

The metabolism of testosterone by Caco-2/TC7 cells in culture was used to study the interaction of acetoxy stilbenes with CYP3A4. All of the examined acetoxy stilbenes inhibited the metabolism of testosterone in a dose-dependent manner, significantly different from control, and maximal inhibition was achieved by application of 50 μM (Fig. 4). However, TAR had the weakest inhibition capacity of all of the tested compounds. Expression of the results of the application of 10 μM of each compound as a percentage of the control revealed a similar trend, in which the order of inhibition capacity was DAR > MAR > TAR, with calculated rates of 45%, 24% and 10%, respectively (Fig. 5). Resveratrol exhibited a stronger inhibition capacity than all of the acetoxy stilbenes, with 10 μM of resveratrol decreasing the enzymatic oxidation of testosterone by more than 80% after 2 h of incubation. This inhibition was similar to that observed for ketoconazole, a well-recognized specific inhibitor of CYP 3A (Fig. 5). After 2 h of incubation, the inhibition rate was reduced in all treatments, but was still significantly lower than the control measured at each incubation time.

### Inhibition of microsomal-CYP3A activity by acetoxy stilbenes

**Figure 6:**
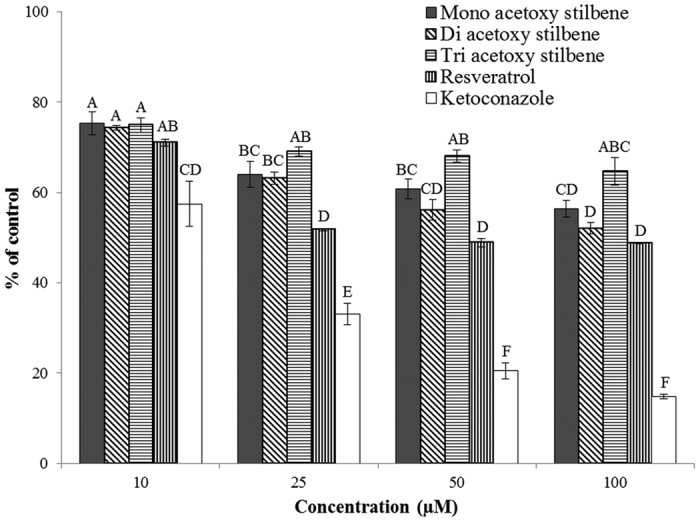
Inhibition of CYP3A-mediated formation of tritiated water. Reduction in microsomal CYP3A activity in the presence of different concentrations of the acetoxy analogues, resveratrol and ketoconazole. Results are expressed as the percentage of the activity of the control. Data are means ± S.E. of three replicates. Different letters indicate significant differences at *P *< 0.05, as determined by ANOVA, followed by the Tukey-Kramer test.

All of the tested stilbenes were shown to inhibit the microsomal CYP3A activity, measured as a reduction in the release of tritiated water through the hydroxylation of testosterone [1,2,6,7-^3^H(N)] (Fig. 6). Inhibition rates, expressed as a percentage relative to the control, indicated that the strongest inhibition was observed following the application of ketoconazole, which had an IC_50_ value of 14 μM. In this system, the IC_50_ of resveratrol, as estimated from concentration–response curves, was found to be 28 μM. The IC_50_ values for MAR, DAR and TAR in this system could not be calculated due to the low levels of inhibition induced by those substances. In agreement with the results obtained for the TC7 cells, TAR showed the lowest inhibition capacity and the application of 10 μM of TAR resulted in the same degree of inhibition as 100 μM. DAR and MAR demonstrated similar potencies when applied at a concentration of 100 μM, their potencies (44% and 48% inhibition, respectively) were similar to that of resveratrol (Fig. 6).

### Computational analysis

**Figure 7:**
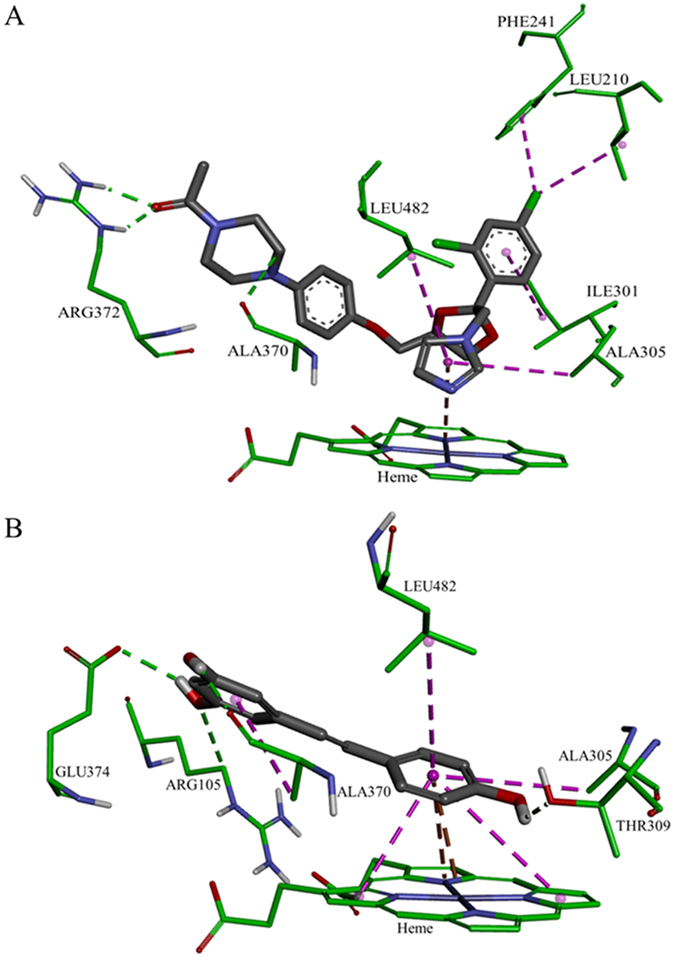
Docking results of the ligands at the binding site of CYP3A4 showing the interacting residues: ketoconazole (**A**, CDOCKER energy = −40.752 kcal/mole; CDOCKER interaction energy = −60.612 kcal/mole) and resveratrol (**B**, CDOCKER energy = −25.504 kcal/mole; CDOCKER interaction energy = −33.960 kcal/mole). Ligands are shown as gray sticks and receptor residues are shown as green sticks. Bonds are shown as dashed lines color-coded as follows: hydrophobic interactions in magenta, electrostatic interaction in brown and hydrogen bonds in green.

**Figure 8:**
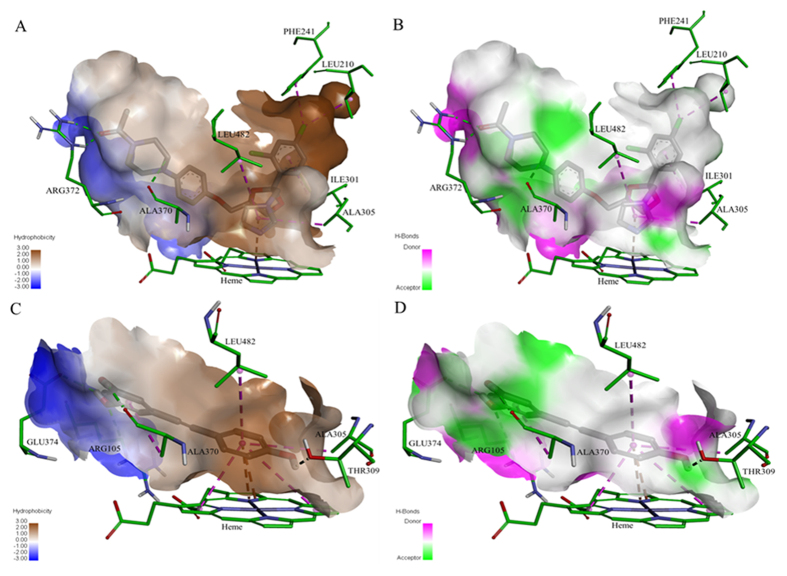
Maps of the hydrophobicity (**A,C**) and hydrogen bonds (**B,D**) at the binding site of CYP3A4 docked with the different ligands: ketoconazole (**A,B**); resveratrol (**C,D**). Ligands are shown as gray sticks and receptor residues are shown as green sticks.

The orientation and residue bonding of ketoconazole inferred from the X-ray structure of human CYP3A4 (PDB code 2V0M) served as a model for computing the interactions of resveratrol and the molecules synthesized here. To validate the suitability of the selected docking model, we first docked ketoconazole and ensured that its bonding to the CYP3A4 binding site was restored to the initial state, as in the original 2V0M structure. According to the ketoconazole-docking simulation, the binding site can be described in terms of three main features: a solid hydrophobic region, H-bond donors/acceptors and electrostatic interactions. The hydrophobic cluster includes the alkyl and alkyl-pi interactions between CYP3A4 residues Leu210, Phe241, Ile301, Ala305 and Leu482, and the chlorinated aromatic ring and imidazole ring of the ketoconazole molecule. The main H-bond donor residue is CYP3A4 Arg372, which interacts with the ketonic oxygen atom of ketoconazole; whereas the oxygen atoms of Ala370 serve as H-bond acceptors. The nitrogen atom of the heme group provides further anchoring to the molecule through its electrostatic interactions (anion-pi) with the imidazole ring of the ketoconazole (Figs 7A and 8A,B).

Among the tested ligands, ketoconazole had the highest docking score with the lowest CDOCKER energy (−40.752 kcal/mole, Fig. 7A). Resveratrol interacted with the enzyme much like ketoconazole did, but the resveratrol-enzyme interaction involved additional interactions at the binding site. Since resveratrol is smaller than ketoconazole, it is incapable of carrying out a significant part of the hydrophobic interactions, such as those between ketoconazole and residues Leu210, Phe241 and Ile301. On the other hand, the B ring of resveratrol can engage in additional hydrophobic and electrostatic interactions at the binding site. We found alkyl-pi interactions between the B ring of resveratrol and residues Ala305 and Leu482, as well as stacked pi–pi interactions with two pyrrole rings of the heme group; whereas cation-pi and anion-pi interactions were observed with the heme iron and nitrogen, respectively, and additional hydrophobic interaction was observed between the A ring and Ala370. The hydrogen atoms of the three hydroxyl groups of resveratrol act as H-bond donors and bind Ala370 and Glu374 (ring A), and Thr309 (ring B; Figs. 7B and 8C,D). The overall CDOCKER energy computed for resveratrol was −25.501 kcal/mole (Fig. 7B), indicating that resveratrol has a weaker affinity for the enzyme than ketoconazole does, in agreement with the results of our *in vitro* inhibition assay.

**Figure 9:**
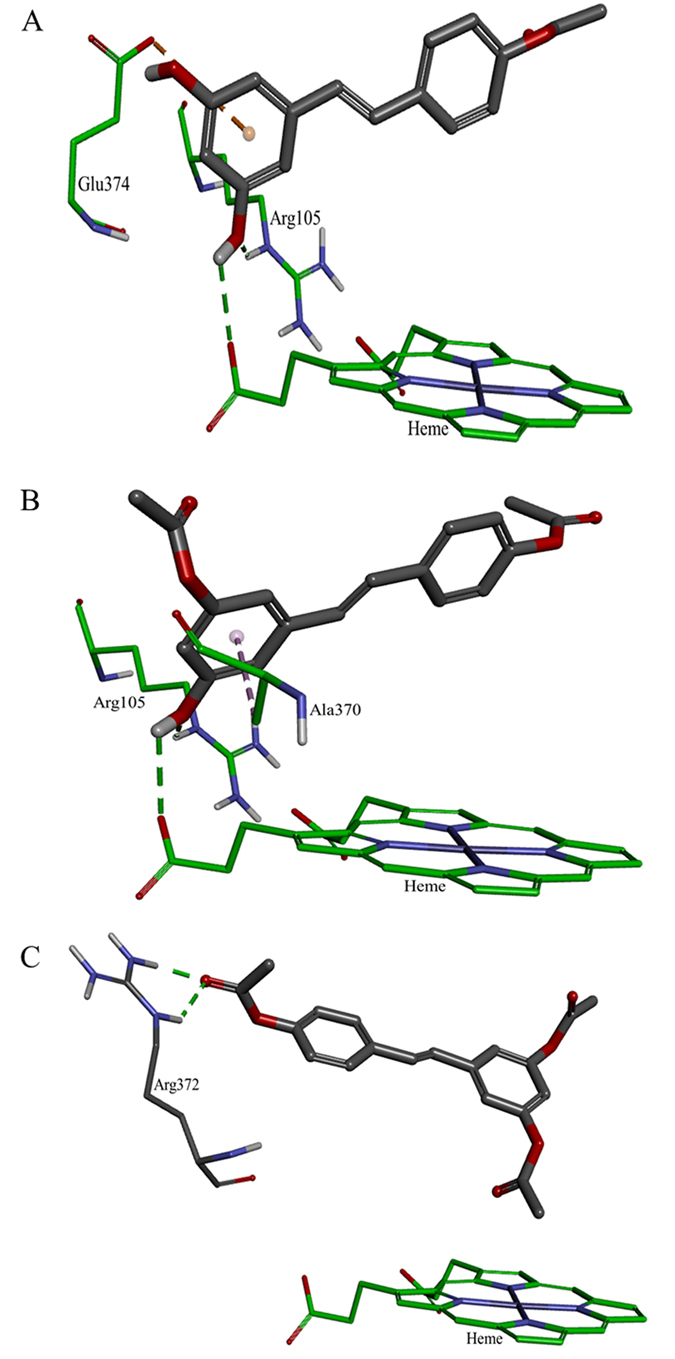
Docking results of the ligands at the binding site of CYP3A4 showing the interacting residues: MAR (**A**, CDOCKER energy = −21.741 kcal/mole; CDOCKER interaction energy = −29.358 kcal/mole), DAR (**B**, CDOCKER energy = −25.733 kcal/mole; CDOCKER interaction energy = −34.376 kcal/mole) and TAR (**C**, CDOCKER energy = −29.487 kcal/mole; CDOCKER interaction energy = −36.707 kcal/mole). Ligands are shown as gray sticks and receptor residues are shown as green sticks. Bonds are shown as dashed lines color-coded as follows: hydrophobic interactions in magenta, electrostatic interaction in brown and hydrogen bonds in green.

**Figure 10:**
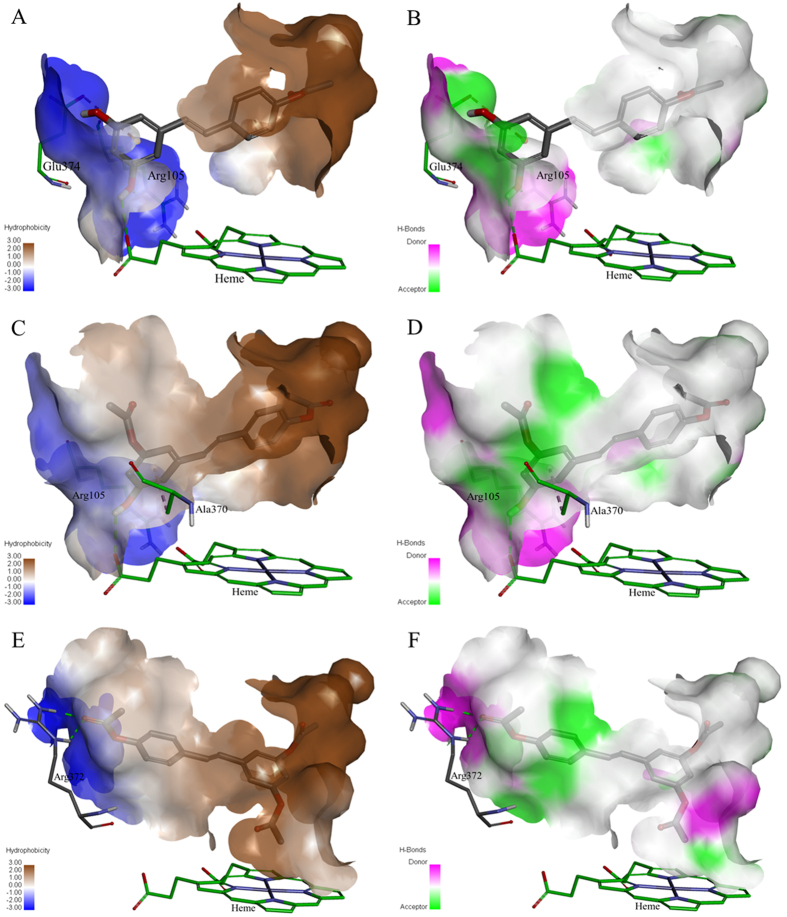
Maps of the hydrophobicity (**A,C,E**) and hydrogen bonds (**B,D,F**) at the binding site of CYP3A4 docked with the different ligands: MAR (**A,B**), DAR (**C,D**) and TAR (**E,F**). Ligands are shown as gray sticks and receptor residues are shown as green sticks.

The acetoxy stilbenes docking at the active-site cavity demonstrated a different arrangement, departing from the heme group and consequently producing remarkably fewer interactions (in all categories), as compared to resveratrol (Figs 9 and 10). In the cases of MAR and DAR, the hydroxyl group at position 5 in the A ring interacted via H-bonds with CYP3A4 Arg105 and the heme (Figs 9A,B and 10A–D). Further anchoring of MAR and DAR involved electrostatic (pi-anion) interaction with Glu374 and hydrophobic (pi-alkyl) interaction with Ala370. Despite the decreased interactions of the acetoxy stilbenes at the binding site, their affinity for CYP3A4 was relatively high, as compared to resveratrol, as demonstrated by the CDOCKER energy values. DAR ranked in the same level with resveratrol (−25.733 and −25.504 kcal/mole, respectively) and MAR placed below it, with the highest value (−21.741 kcal/mole, Fig. 9). Interestingly, the CDOCKER energy of TAR, which had the weakest inhibitory effect on the enzyme, was lower than that of the other acetoxylated analogues and even that of resveratrol itself (−29.487 kcal/mole, Fig. 9). Our use of the Ligand Interaction tool revealed that TAR docks in an opposite and distal orientation, as compared to the other stilbenes. As a result, it does not interact with the heme group (Figs 9C and 10E,F). The ketonic oxygen atom of the acetoxy group at the 4′ position of TAR serves as an H-bond acceptor for the distal CYP3A4 Arg372.

## Discussion

In recent studies, evidence has accumulated to indicate that there are potent interactions of clinical importance between CYP3A4 and dietary polyphenols, including flavonoids, phenolic acids, phenolic alcohols, stilbenoids and lignans^4^. The inhibitory effects of *t*-resveratrol on CYP3A4, both *in vitro* and *in vivo*, are well documented in the literature^15^,^16^,^17^,^18^,^19^,^37^ and clinical trials have found that the administration of resveratrol increases plasma concentrations of several drugs^20^,^27^. Several QSAR and pharmacophore mapping studies point to the importance of lipophilicity, and also to the role of H-bonds in determining how various molecules interact with CYP3A4^28^,^29^,^31^,^33^.

A previous study described the effect of a molecule’s lipophilicity on its interactions with CYP3A4 using synthetic modifications of resveratrol. That study found that methoxy-stilbenes have lower IC_50_ values and greater affinity for CYP3A4, as compared to the parent resveratrol and its glucosides^17^. Our recent work revealed that the introduction of an aldehyde group to the A ring of resveratrol decreases its ability to inhibit CYP3A4, as demonstrated using *in vitro* and *in silico* models. This finding highlights the importance of the electrostatic interactions of the molecule at the active site of the enzyme, in addition to hydrophobic and H-bonds^37^. Unlike a hydroxyl group, an acetoxy group is large and is a strong H-bond acceptor^39^. Thus, gradual substitution of hydroxyl groups with acetoxy groups may not only increase the lipophilicity of a molecule but also introduce H-bond acceptors to the molecule. Here, acetoxylated stilbenes served to differentiate lipophilicity from structure-activity relationships in inhibiting CYP3A4, using two different biological models and simulation software.

The CYP3A4-mediated metabolism of testosterone using Caco-2/TC7 cells was designed to last 8 h, to optimally represent passage through the intestine. A linear curve was observed over a 2 to 6 h incubation period, leading to the selection of this time interval for the study the inhibition of human intestinal CYP3A4. We monitored the substrate consumption and, in this context, the treatments were significantly different from the control throughout all of the examined incubation periods. Resveratrol inhibited CYP3A4 in a manner similar to that of ketoconazole, with calculated IC_50_ values in agreement with those presented in previous works^16^,^40^,^41^,^42^,^43^. Increasing the lipophilicity to achieve higher inhibition proved right for the transformation of MAR into DAR, but not for resveratrol into MAR, or for the additional acetylation to yield TAR. Indeed, we could not calculate IC_50_ values for the acetoxy stilbenes since we could not achieve sufficient inhibition in the TC7 cells or in the RLM assay. The above results clearly demonstrate the importance of structural properties in determining the inhibition capacity of closely related polyphenolics against CYP3A4.

In a recent study, examination of the docking of ketoconazole, resveratrol and resveratrol-aldehyde at the binding site demonstrated the role that hydrophobic and electrostatic interactions play in the interactions of polyphenolics with CYP3A^37^. Similarly, computational tools served to describe the interactions of food colorants with human serum albumin^44^. Here, using a docking simulation, we showed that ketoconazole is involved in strong hydrophobic interactions at the active site, in addition to its electrostatic interaction with the heme and a few H-bonds (Figs 7A and 8A,B). The importance of the lipophilicity of the CYP3A4-ligand and its ability to establish hydrophobic interactions are well documented^4^,^17^,^31^. Resveratrol has fewer hydrophobic interactions than ketoconazole, which may explain its higher CDOCKER energy. However, resveratrol does have strong hydrophobic and electrostatic interactions with the heme group and its iron/nitrogen atoms, and H-bonds with more residues than ketoconazole at the active site (Figs 7B and 8C,D). The role of the heme-iron in the metabolic mode of action of CYP enzymes is well established^45^,^46^,^47^,^48^, as is the contribution of electrostatic interactions with the heme group to ligand affinity for CYP3A4^37^.

Docking of the acetoxy analogues at the enzyme cavity was shown to produce a new array of bonds, i.e. two hydrogen bonds with Arg372 and the B ring of TAR, resulting also in an increased distance from the heme prosthetic group (Figs 9 and 10). Despite the hydrophobicity of the acetoxy group at the reactive 4′ position, the mono- and di-acetoxy stilbenes (i.e., MAR and DAR) did not interact with CYP3A4 residues via this moiety of the molecule. Rather, the hydroxyl groups at position 5 interacted with Arg105 and the heme. However, DAR is anchored more closely than MAR above the heme group via a hydrophobic (pi-alkyl bond) with Ala370. This may explain the higher affinity and inhibition capacity of DAR, as compared to MAR. Tri-acetoxy stilbene apparently has a strong affinity for the enzyme, as demonstrated by the docking score. In fact, its CDOCKER energy was lower than those of other stilbenes, including resveratrol. Values of CDOCKER energy shown here is the estimated sum of interaction energy and strain energy, and was in complete agreement with values of CDOCKER interaction energy. Calculating the strain energy as the difference between CDOCKER energy and CDOCKER interaction energy yielded proximate values for all stilbenoids studied here with the lowest for TAR. A much higher value was calculated for ketoconazole. The low value for TAR may also be due to the formation of only two H-bonds with CYP3A4, and no additional electrostatic or hydrophobic interactions.

The results of the computational analysis suggest that TAR is inserted in an opposite orientation, in which, unlike previously studied stilbenes, the A ring faces the hydrophobic region of the binding site and is distally above the heme group (Figs 9C and 10E,F). In addition, no potential interaction between TAR and the heme was detected in the software simulation. Based on the essential involvement of the heme-iron in the catalytic mechanism of the enzyme^45^,^46^,^47^,^48^, we suggest that the binding of TAR at a site distal from the heme may explain the low level of *in vitro* inhibition of the enzyme by this molecule. Further and following its insertion into the active-site cavity, TAR may introduce a new set of available functional groups for secondary substrates to interact with, rather than inhibit enzyme activity via direct interaction with the catalytic site of CYP3A4. The latter suggestion could be examined by simultaneous insertion of two ligands into the cavity, but one should note that even the simultaneous insertion of two identical molecules dramatically decreases the degrees of freedom allowed in by nature, e.g. in the sense of order and orientation of approach, and the presence of other substrates or strongly bound water molecules. However, and unfortunately, that option is not available in the present simulation software.

A simulation of the docking of acetoxylated stilbenes, resveratrol and ketoconazole at the active site of CYP3A4 provided a mechanistic explanation of the lower inhibition caused by the more hydrophobic compounds, showing that the replacement of a hydroxyl group on the aromatic rings leads to a large loss of hydrophobic, H-bond and electrostatic interactions. Interestingly, the CDOCKER energy values of the acetoxy stilbenes were found to be relatively low, probably due to their low conformational energies. The molecules synthesized here, together with the computer simulation, allowed us to reveal a unique behavior of CYP3A4, which may explain its documented behavior involving a very wide spectrum of substrates. Our findings suggest the option of a simultaneous insertion of two molecules into the active site, which adds to reports that some molecules with a strong affinity for the binding site change the properties of that binding site and actually “open” it for additional molecules^49^. This change is also accompanied with a new set of requirements as they relate to the ability of new molecules to produce interactions with the modified active site. In this sense, TAR serves as a modifier of the active site in a manner similar to changing an amino acid residue at the active site using molecular tools. This is clearly demonstrated for the tri acetoxy-stilbene (TAR), which despite its calculated high affinity to the binding site (probably due to its high hydrophobicity), is not likely to establish any interaction with the catalytic site of the enzyme. This explains the low inhibition capacity of this molecule *in vitro* and highlights the importance of the ligand’s proximity to the heme group of CYP3A4.

In conclusion, this work demonstrates for the first time that a substrate-induced modification of the active site of CYP3A4 can be predicted using simulation models. These predictions are in agreement with results achieved using crystallography for an abundant drug, e.g. erythromycin, and its interactions with CYP3A4^49^. Indeed, the low inhibition capacity of TAR in both a human cell line and rat liver microsomes may be explained by these simulation results. Consequently, such results may provide a unique illustration of the importance of proximity of the ligand to the active site of the enzyme. In all, the use of software based predictions together with a comprehensive knowledge of the enzyme’s mode of action and biological assays, allow a deeper understanding of complex enzyme-substrate interactions.

## Methods

### Standards and chemicals

*t-*Resveratrol (trans-3,4′,5-trihydroxystilbene, 99%), testosterone (99%) and ketoconazole (98%) standards were purchased from Sigma Aldrich, Israel. Testosterone [1,2,6,7-^3^H(N)] was purchased from (Perkin Elmer, USA). All other chemicals and reagents were purchased from Sigma Aldrich, unless otherwise noted.

### Acetylation of resveratrol

The synthesis of acetoxylated analogues of resveratrol was performed using acetylation reactions, as described by^50^. A solution of resveratrol (0.6 g, 2.63 mmol), acetyl chloride (0.56 mL, 7.88 mmol) and triethelamine (1.009 mL, 7.88 mmol) was stirred in a 50-mL round-bottom flask with acetone (20 mL) acting as a solvent. After the solution was stirred overnight, it was acidified (<5) using hydrochloride acid (2N). The crude product was extracted in the organic layer using ethyl acetate (3 × 20 mL). Sodium hydrogen carbonate (20 mL) was used to remove excess hydrochloric acid. The product was dried using magnesium sulfate and then gravity-filtered. The crude product was cleaned over a silica gel column (ethylacetate:hexane 3:7), yielding three different acetoxylated products: 70 mg of mono-acetoxy resveratrol (yield: 10%), 33 mg of di-acetoxy resveratrol (yield: 4%) and 207 mg of tri-acetoxy resveratrol (yield: 22%). ^1^H NMR and ^13^C NMR spectra of the products were obtained with a 200-MHz Bruker NMR spectrometer (Bruker Biospin GmbH, Rheinstetten, Germany).

### NMR analysis

#### Mono-acetoxy resveratrol (MAR)

(4′-acetoxy-3,5-hydroxy-stilbene): ^**1**^**H NMR** (200 MHz, MeOD, ppm): δ 2.26 (s, 3H), 6.21 (t, J = 2.2 Hz, 1H), 6.49 (d, J = 2.2 Hz, 2H), 6.99–7.08 (m, 4H), 7.49–7.54 (m, 2H). ^**13**^**C NMR** (200 MHz, MeOD, ppm): δ 20.9 (CH_3_), 103.2, 106.1, 116.5, 122.9, 128.3, 130.2 (CH), 135.1, 136.6, 148.0, 151.6, 159.7, 171.2, 178.2 (C).

#### Di-acetoxy resveratrol (DAR)

(3,4′-acetoxy-5-hydroxy-stilbene): ^**1**^**H NMR** (200 MHz, MeOD, ppm): δ 2.27 (s, 6H), 6.45 (t, J = 2.2 Hz 1H), 6.78 (q, J = 1.8 Hz, 1H), 6.86 (t, J = 1.8 Hz, 1H), 7.04–7.15 (m, 4H), 7.51–7.57 (m, 2H). ^**13**^**C NMR** (200 MHz, MeOD, ppm): δ 20.9 (CH_3_), 109.3, 111.8, 111.9, 124.8, 127.6, 129.2, 129.5 (CH), 136.9, 140.8, 151.7, 153.4, 157.1, 159.6, 171.1 (C).

#### Tri-acetoxy resveratrol (TAR)

(3,4′,5-acetoxystilbene): ^**1**^**H NMR** (200 MHz, MeOD, ppm): δ 2.30 (s, 9H), 6.83 (t, J = 2 Hz, 1H), 7.07–7.26 (m, 6H), 7.56–7.61 (m, 2H). ^**13**^**C NMR** (200 MHz, MeOD, ppm): δ 20.9 (CH_3_), 115.7, 118.1, 120.4, 123.1, 128.1, 128.7 (CH), 130.7, 137.7, 151.9, 152.9, 170.7, 171.1, 174.8 (C).

#### Maintenance of the Caco-2/TC7 cells

Caco-2/TC7, a subclone of Caco-2, expresses a high level of CYP3A4 enzyme, which is quite common in the human intestine^51^,^52^,^53^. The TC7 cell line is commonly used to study CYP3A4-mediated metabolism^54^,^55^,^56^. The Caco-2/TC7 cells used in this study were a gift from Dr. M. Rousset (INSERM U505, Paris)^52^. Throughout the experiment, these cells were maintained under aseptic conditions in Dulbecco’s modified Eagle medium (DMEM) at 37 C with 5% CO_2_ and 95% air. The DMEM was supplemented with 20% fetal bovine serum, 100 units/mL penicillin (Biological Industries, Israel), 100 μg/mL streptomycin (Biological Industries), 1% non-essential amino acids solution (concentrated 100X, Biological Industries) and 1% L-glutamine solution (200 mM, Biological Industries). The medium was pre-warmed to 37 C prior to contact with the cells. The Caco-2/TC7 cells were used between passages 25 and 35 and grown in 75-cm^2^ flasks. New flasks were seeded at a concentration of 3–4 × 10^4^ cells/cm^2^.

#### Testosterone metabolism in Caco-2/TC7 cells

Caco-2/TC7 cells were seeded at 2–3 × 10^4^ cells/cm^2^ in 12-well plates. The medium was changed every second day and the cells were allowed to grow and differentiate for up to 14 days after confluence was reached. On the day of the experiment, testosterone, ketoconazole, resveratrol, MAR, DAR and TAR were dissolved in methanol, sonicated for 10 min (Transsonic T420/H, Elma, Germany) and then added to the treatment medium (maximum methanol concentration in the medium was 0.1%). Testosterone, used as a substrate of CYP3A4 enzyme, was added to each cell well in all of the treatments at a final concentration of 200 μM. The actual concentration of testosterone in the reaction medium at time 0 was 31 μM, as determined using the below described HPLC method. The treatments in this study were as follows: Control 1 included only testosterone incubated with Caco-2/TC7 cells and Control 2 included a specific inhibitor of CYP3A4, ketoconazole (10 μM). The stilbene treatments included resveratrol (10 μM) and the acetoxy analogues MAR, DAR and TAR at different concentrations (10, 25, 50, 100 μM). All treatments were carried out for three incubation periods, 2, 4 and 6 hours (in addition to time 0), and there were three replicates of each treatment/incubation time combination. At the end of incubation, the culture medium was transferred to Eppendorf vials, the cell were harvested by scraping after addition of HPLC-grade methanol (200 μl) and then transferred to the Eppendorf vials. Then, 100 μl of HPLC-grade acetonitrile was added and the Eppendorf vials were shaken vigorously and then, centrifuged at 14,000 RPM for 5 min (SIGMA Laborzentrifugen, Germany). The supernatant was then filtered through 0.22 μm filter into HPLC vials.

#### HPLC analysis

High pressure liquid chromatography (HPLC) analysis was carried out on a Surveyor HPLC system (Thermo Finnigan, USA) using a RP-C18 Luna column 250 mm × 4.6 mm × 5 μm (Phenomenex, USA). Elution was performed with water (Solvent A) and acetonitrile (Solvent B) at a flow rate of 0.7 mL/min. The mobile phase gradient was modified as follow: B in A (v/v), from 5–25% for 5 min, from 25–75% for 10 min, from 75–95% for 5 min, followed by holding for 2 min, dropped again to 5% for 1 min and held for 4 min. Samples were examined with a photodiode array detector at 244 nm for testosterone and its metabolite, and at 280 nm and 306 nm for resveratrol and its derivatives. Peaks were scanned between 196 and 450 nm.

#### Cell viability assay

The effects of the acetoxy stilbene analogues on the proliferation of Caco-2/TC7 cells were assessed by the MTT assay. Cells were seeded onto 96-well plates at a density of 10^4^ cells per well and incubated for 24 h. Then, the cells were treated with DMEM (control) and the acetoxy stilbene analogues MAR, DAR and TAR at different concentrations (5, 10, 25, 50, 100 μM) for an additional 8 h. Cells were incubated with 3-(4,5- dimethylthiazol-2-yl)-2,5-diphenyl tetrazolium bromide (MTT, 0.5 mg/mL) for 1 h and there was a follow-up incubation with DMSO for 20 min. The formation of the colored formazan dye was assessed colorimetrically at 550 nm in an ELX 808 Ultra microplate reader (BIO-TEK Instruments, London, UK) using KCJunior software (York, UK).

#### Preparation of rat liver microsomes

Male rat liver microsomes (RLM) were prepared from adult male Sprague-Dawley rats (175–199 g), according to published protocol with minor modifications^57^. All experiments were conducted in accordance with the Animal Care and Use Guidelines of the Hebrew University and were approved by the National Research Council for Care and Use of Laboratory Animals. Five livers were harvested from rats, weighed, frozen in liquid nitrogen, minced, and stored at −80 °C until use. Upon usage, minced livers were perfused with ice-cold homogenization buffer (50 mM Tris, pH 7.5, 150 mM KCl) at a ratio of 5 mL homogenization buffer to 1 g liver. Phenyl methyl sulfonyl fluoride (1 mM) was added right before homogenization. Livers were homogenized using a motorized homogenizer. The homogenate was centrifuged at 10,000 g for 10 min at 4 °C. The supernatant was collected and centrifuged again at 100,000 g for 70 min at 4 °C to yield microsomal pellets. Microsomes were re-suspended in ice-cold washing buffer (100 mM tetra-sodium pyrophosphate and 10 mM EDTA, pH 8.8) and re-pelleted by centrifugation at 100,000 g for 70 min at 4 °C to yield microsomes. The microsomal pellets were then re-suspended in ice-cold Tris buffer (50 mM, pH 7.5) containing 50% glycerol, aliquoted into vials (0.2 ml per vial) and stored at −80 °C until use. Microsomal protein concentration was determined using a Bradford protein assay^58^ calibrated using bovine serum albumin.

#### Testosterone metabolism by microsomal CYP3A

The interaction of CYP3A with testosterone was examined using a tritiated water assay adapted from^59^. Reactions were carried out in 96-well plates (Nunc, Roskilde, Denmark) and all samples were assayed in triplicates. RLM were thawed on ice and 100 μg was added to each well, along with reaction buffer (10 mM K_2_HPO_4_, 100 mM KCl, 1 mM EDTA, 1 mM dithiothreitol, pH 7.4) containing 8 nM testosterone [1,2,6,7-^3^H(N)] and 1.5 mM NADPH. Resveratrol, acetoxy stilbene analogues and ketoconazole were first dissolved in 30% methanol and sonicated, and then stock solutions were prepared in reaction buffer (maximum methanol concentration in each well was 0.95%). The different treatments were added to the reaction wells to achieve final concentrations of 10, 25, 50 and 100 μM. All treatments were assayed in triplicate with the following controls: a buffer control containing the reaction buffer instead of the inhibitor (no treatment); a methanol control containing 0.95% methanol instead of the inhibitor, to account for possible effects of this solvent; and a RLM control that did not contain any microsomes, to provide a background value. Reactions were then incubated for 4 h with continuous shaking at the optimal temperature of 37 °C. To stop the reaction, 1 mL of ethyl ether was added, followed by vortex-mixing. Ether and aqueous layers were allowed to separate for 10 min and then transferred to −80 °C for 15 min, to completely freeze the aqueous phase. The aqueous phase was left in the chemical hood for 1 h, to allow the complete evaporation of ether traces. The aqueous phase was shaken with 2 volumes (400 μl) of dextran coated (0.5%) charcoal slurry (5% coated charcoal in phosphate buffer) for 1 min and centrifuged at 3000 g at 4 °C for 5 min. The supernatant (300 μl) was collected into scintillation vials and mixed with 5 mL scintillation fluid. ^3^H was measured as disintegration per 5 min using a liquid scintillation β counter (Canberra-Packard Tri-Crab 1600, CA).

#### Computational modelling and simulation software

The crystal structure of human CYP3A4 determined with ketoconazole^49^, available in the protein data bank (PDB entry 2V0M), was used for the protein-modelling in this study. Ligand-structure sketches were obtained using ChemDraw Ultra 8.0 and the energy minimization of ligands was achieved with Chem3D Ultra 8.0 (CambridgeSoft Corporation, USA). Ligands in an MDL molfile were uploaded into Discovery Studio 4.0 (Accelrys, San Diego, CA) and that program’s Clean Geometry tool was used to minimize the structure energy. Conformations were then generated using the Generate Conformations protocol and the Best method (other parameters were kept at the default values). 2V0M represents a tetrameric crystal structure of CYP3A4 that includes four chains (A-D). Chain D was chosen for the simulations based on the isotropic displacement property. The second molecule of ketoconazole, which was identified in an antiparallel orientation above the first ketoconazole molecule, was removed, as were the extracellular and intracellular loops, since they were not predicted to be part of the binding site. Prior to analysis, the iron atom in the heme group was constrained to preserve its bonding to the nitrogen atoms of the heme after the application of the CHARMm force field. The protein was prepared using the Prepare Protein protocol and a binding site was created using Define and Edit Binding Site protocol (default parameters were used). Each ligand conformation that was generated was superimposed on ketoconazole from the 2V0M, according to the alignment specification (80% steric and 20% electrostatic), and the top five conformations with the highest degree of similarity to ketoconazole were chosen for the docking simulations. Docking of the ligands (ketoconazole, resveratrol, MAR, DAR and TAR) was performed using the CDOCKER protocol. After the docking, analysis of the ligand interactions at the binding site was performed using the Ligand Interactions tool. A final analysis was performed manually. All protocols and tools are available as part of the Discovery Studio 4.0 (Accelrys).

## Additional Information

**How to cite this article**: Basheer, L. *et al*. Inhibition of cytochrome P450 3A by acetoxylated analogues of resveratrol in *in vitro* and *in silico* models. *Sci. Rep.* **6**, 31557; doi: 10.1038/srep31557 (2016).

## Figures and Tables

**Figure 1:**
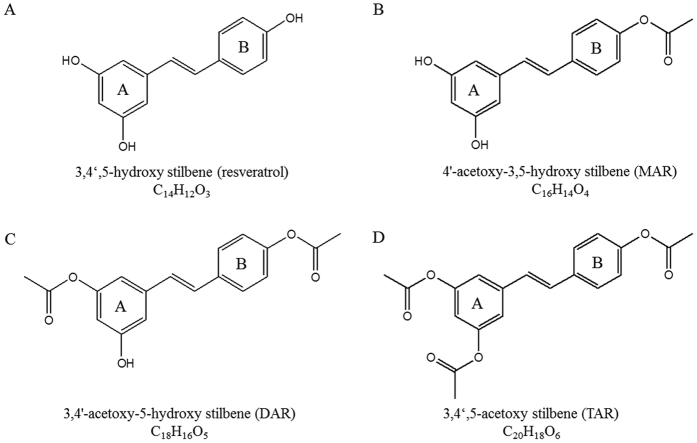
Chemical structures of resveratrol (**A**), mono-acetoxy resveratrol (**B**), di-acetoxy resveratrol (**C**) and tri-acetoxy resveratrol (**D**).
